# Thermal Imaging of the Periorbital Regions during the Presentation of an Auditory Startle Stimulus

**DOI:** 10.1371/journal.pone.0027268

**Published:** 2011-11-03

**Authors:** Luke Gane, Sarah Power, Azadeh Kushki, Tom Chau

**Affiliations:** 1 Institute of Biomaterials and Biomedical Engineering, University of Toronto, Toronto, Ontario, Canada; 2 Bloorview Research Institute, Toronto, Ontario, Canada; University of Granada, Spain

## Abstract

Infrared thermal imaging of the inner canthi of the periorbital regions of the face can potentially serve as an input signal modality for an alternative access system for individuals with conditions that preclude speech or voluntary movement, such as total locked-in syndrome. However, it is unknown if the temperature of these regions is affected by the human startle response, as changes in the facial temperature of the periorbital regions manifested during the startle response could generate false positives in a thermography-based access system. This study presents an examination of the temperature characteristics of the periorbital regions of 11 able-bodied adult participants before and after a 102 dB auditory startle stimulus. The results indicate that the startle response has no substantial effect on the mean temperature of the periorbital regions. This indicates that thermography-based access solutions would be insensitive to startle reactions in their user, an important advantage over other modalities being considered in the context of access solutions for individuals with a severe motor disability.

## Introduction

Locked-in syndrome (LIS) is a condition in which the mobility of an individual is severely limited, to the point of complete or near complete paralysis, but cognitive function and awareness are unaffected [1], [2]. The most severe variant of this state, total LIS, renders a person completely immobile [1], [3], [4]. Unaided, individuals with total LIS are unable to communicate or interact with their environment. In these cases, alternative channels of communication can potentially be enabled by systems known as access technologies.

Various technologies, including computer eye-tracking systems and chin or finger switches, have been developed to enable individuals with classical or incomplete LIS to operate purpose-designed communication software, such as scanning keyboards and speech synthesizers [3]. However, there are currently no clinically available interventions for individuals with total LIS. Most research in this area to date has focused on brain-computer interfaces (BCIs). BCIs record signals indicative of brain activity using techniques such as electroencephalography (EEG) and near-infrared spectroscopy (NIRS) [5], [6] and attempt to use these signals to determine the functional intent of the user. Although the results of research into these modalities have been encouraging, there are significant drawbacks. In many cases, these systems require users to be trained to modulate their neurological activity so that the resulting signals can be reliably interpreted [6]. Also, both EEG and NIRS require multiple sensors to be placed directly on the user, which can be time-consuming and can cause discomfort over long periods of wear. Consideration of other access modalities that avoid these challenges is worthwhile.

The use of thermography in the context of access technology is not unprecedented. Thermal imaging has provided an effective access switch for individuals who retain voluntary control over the ability to open and close their mouths by enabling a binary switch triggered by the detection of the warmer tissue exposed by an open mouth [7]–[9]. Infrared thermal imaging also shows promise as a potential access modality for individuals with total LIS. Although psychologically mediated changes in facial skin temperature (such as facial flushing due to embarrassment or discomfort) have been known for some time [10], research has only recently shown that variations in an individual's emotional state produce changes in facial skin temperature that can be reliably detected using thermal imaging [11]. A recent study found that emotional states induced by the viewing of images from the International Affective Picture System could be distinguished from a baseline (i.e., neutral) emotional state using thermal video of the face with an accuracy between 70% and 80% [11]. These findings suggest the potential of an access technology based on infrared thermal imaging to either allow a user to control a computer system through deliberate variations in affective state or to provide caregivers of those with total LIS with insight into their psychological state.

Thermography is an attractive access modality, as it is completely non-contact and, unlike visible light imaging systems, is unaffected by skin colour or ambient lighting conditions. Also, thermography can detect psychological changes using naturally occurring physiological cues that do not require special training to cultivate [11].

However, changes due to involuntary physiological processes that do not represent an individual's intent or emotional state could be problematic for an access solution based on thermal imaging. One mechanism of particular concern is the startle response, i.e., the body's reaction to sudden, unexpected stimuli in the environment. This response is generated by the sympathetic nervous system and is not useful to access systems since it does not reflect a conscious, long-term, or otherwise relevant alteration in mental state. The startle response is known to affect the operation of BCIs by causing false positives [12], [13]; however, it is not known if startling stimuli affect the temperature of the face in a manner that would impact the feasibility of thermal imaging as the foundation for an access system.

The objective of the present study was to further investigate the feasibility of access technology based on infrared thermal imaging by determining if there is a detectable change in the temperature of the inner canthi of the periorbital regions of the face (previously identified as among the most useful regions of the face for thermography-based access [14]) in response to the presentation of an unexpected auditory stimulus. If such a change does exist, any thermography-based access technology would have to include measures to distinguish these startle-induced changes from changes attributable to the user's intent. Failure to do so could undermine the feasibility of using thermal access systems in a practical context, where the likelihood of unexpected, startling events (e.g., a door slamming) is high.

### The Startle Response

The startle response is the response to an unexpected or novel stimulus [15]. The formal startle response in humans is considered to be manifested through blinking of the eyes [16], [17], a generalized motor response in the upper half of the body [16], and changes in blood pressure [18].

Although the startle response can induce changes in the cardiovascular system and blood flow [18], there is minimal literature regarding the measurement of temperature variations (facial or otherwise) in an individual upon the presentation of an unexpected stimulus. One group reports that the presentation of an unexpected auditory stimulus elicits a rapid warming of the periorbital region within 300 milliseconds [19], [20], but the analysis employed in this study was limited. Another paper notes that activity of the sympathetic nervous system (the driving component of the startle response) can direct additional blood flow to the eye and increase the temperature of the skin around the corners of the eyes [21]. Overall, it seems probable that the startle response may affect facial skin temperature as blood perfusion is altered.

## Methods

### Ethics Statement

The experimental protocol was approved by the Research Ethics Board at Holland Bloorview Kids Rehabilitation Hospital. Participants provided written consent after being fully informed of the study protocol.

### Experimental Protocol

Each participant completed 10 sessions. The sessions were approximately 30 minutes in duration and, to counter the effects of habituation, were separated by at least 24 hours. During all sessions, participants were seated in a quiet room (the ambient noise level was approximately 30 dB) facing a thermal camera at a distance of approximately one metre. Where applicable, the participant's external corrective eyewear was removed, as glass is opaque to infrared light and would occlude the regions of interest. Eyewear removal at the start of the equilibration period also avoided any pressure-induced temperature changes on the bridge of the nose and the surrounding tissue. For the first 15 minutes of each session, participants remained seated to allow their temperature to equilibrate to that of the room [22]. The last 15 minutes were used to run experimental trials.

Participants completed 10 trials at each session, yielding a total of 100 trials per participant. Each trial was 30 seconds in duration and successive trials were separated by a one-minute rest period. In half of the trials, the participant was presented with an unexpected auditory stimulus at a randomly selected time. The remaining 50 trials contained no stimulus. The stimulus and non-stimulus trials were randomly interleaved to prevent participants from predicting the occurrence of a stimulus trial.

Throughout all trials, the participant performed a simple image-matching task. This task was used to reduce possible fear potentiation caused by anticipation of the startle stimulus [17]. During this task, randomly chosen pairs of images from one of 10 different image sets (each containing five different images) were presented for 1.5 seconds (i.e., 20 image pairs were displayed per trial). The participant was instructed to press a large, hand-held button when the images matched; this button was also used to begin each trial after the one minute inter-trial delay had elapsed.

### Participants

Eleven adult participants (9 female, mean age 30.2±10.8 years) were recruited from the population of staff and students at the Bloorview Research Institute within the Holland Bloorview Kids Rehabilitation Hospital. Participants were required to be in good health and to possess sufficient visual acuity to comfortably perform the image-matching task described above without the aid of external corrective eyewear. Participants with any conditions that could result in an adverse reaction to the startling auditory stimulus (e.g., a social anxiety disorder or cardiovascular condition) were excluded. To comply with ethics requirements, participants were informed that the study would include the randomized presentation of an auditory startle stimulus before consenting to the study.

### Instrumentation and Recorded Data

**Figure 1:**
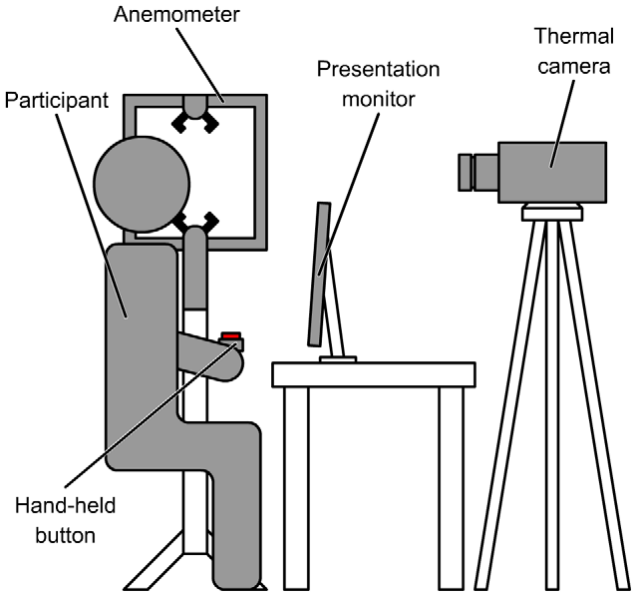
Configuration of experimental apparatus.

During each trial, a thermal video of the participant's head was recorded using a FLIR SC640 thermal camera. Data from this instrument consisted of a sequence of timestamped frames composed of temperature pixels i.e., pixels whose values were temperature readings in units of Kelvin (a measurement in Kelvin can be converted to degrees Celsius by subtracting 273.15). Temperature readings were recorded at a resolution of 0.1 mK. The measurement range of the camera was set to −40°C to 120°C for all recordings (it should be noted that this is one of three default temperature ranges for this camera and the relatively large measurement range associated with this setting does not compromise the resolution of temperature recordings as may be the case with older units). Thermal video was recorded at 15 Hz with a resolution of 640×480 temperature pixels. To ensure an unobstructed frontal view of the participant's head, the thermal camera was mounted on a tripod above and just behind the screen on which the task images were displayed (see Figure 1).

Ambient temperature and airspeed near the participant were also recorded throughout each trial at a frequency of 1 Hz using an R.M. Young model 81000 ultrasonic anemometer. This permitted the detection of changes in the ambient environmental conditions (e.g., due to the building's heating/cooling system, which could not be controlled by the experimenter) that could have potentially affected the thermal data. The anemometer was positioned to the left of the participant just above eye level.

Immediately before each trial, the thermal camera was calibrated for ambient room conditions, specifically temperature and relative humidity. Ambient temperature was automatically acquired prior to each trial using the anemometer. Ambient relative humidity was read manually from an analog hygrometer at the beginning of each session. If, during a session, the relative humidity was observed to have changed by more than 1% from its initial value, this value was manually updated.

All recorded data were synchronized and timestamped relative to the start of each trial using a custom data acquisition program developed in LabVIEW. This program was also used for the randomization and presentation of the task images and the auditory startle stimulus. The LabVIEW program also recorded the times at which the auditory stimulus was presented.

### Auditory Stimulus

The unexpected auditory stimulus was a 2 kHz square-wave tone, 50 ms in duration, with instantaneous rise and fall times. The stimulus was presented through generic stereo computer speakers. The presentation volume was 102 dB, as measured by a decibel meter held at the approximate location of a participant's head. These parameters were chosen based on existing precedent for auditory stimuli in studies examining the startle response [16], [23].

For trials that included the presentation of the stimulus, its presentation time relative to the start of the trial was selected randomly such that it occurred no earlier than two seconds into the trial and no later than seven seconds before the end of the trial. The randomized presentation of the auditory stimulus, both between and within trials, was intended to reduce the effects of habituation.

### Thermal Data Extraction

Temperature data were extracted from the thermal video collected during each trial. The cross-search algorithm [24] was used to track the left and right inner canthi of the periorbital regions of the participant's face throughout each trial. The mean squared error (MSE) was used as the matching criterion, as it produced better tracking results in preliminary analysis of the data than the recently developed structural similarity index [25]. The reference regions for the tracking algorithm were specified on the first frame of each thermal video except for trials in which the participant pressed the hand-held button to begin the trial before moving his or her head to face the camera. In these cases, the first frame in which the participant was fully facing the camera was used to provide reference regions and the tracking began at that frame. For each thermal video, the location and size of the reference regions for each periorbital were set manually before starting the tracking process to account for variation in the size of the inner canthus of the periorbital region among participants and minor changes in the distance between their faces and the thermal camera lens. Both regions were programmed to be the same size for a given trial. The mean tracking region dimensions across all usable trials were 27.0±3.9 by 38.5±6.0 pixels.

This tracking procedure resulted in two sets of 2-dimensional temperature time series data (one for each periorbital region) per trial. For each trial, a mean temperature time series was obtained for each region by taking the upper 50% trimmed mean of that region's temperature values for each frame of the trial's thermal video. This robust estimator was chosen to reduce the effect of variation in the lower temperature values around the border of the tracking region and focus on the warmer temperature pixels associated with blood perfusion in the inner canthus of the periorbital region [11]. Variation in the border values was attributable to slight changes (on the order of a few pixels) in the position of the tracking region relative to the centre of the periorbital region during the tracking process.

**Figure 2:**
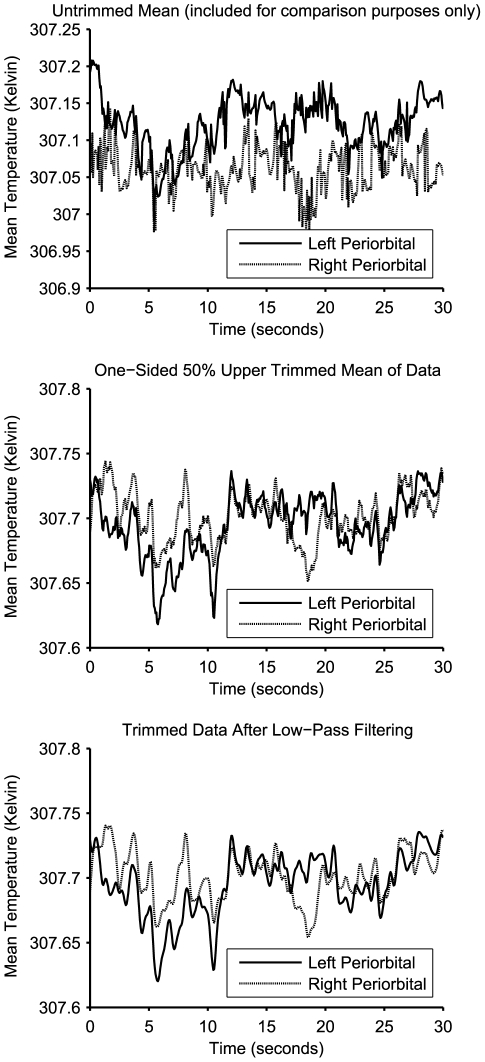
A visual comparison of the mean temperature data at each pre-processing stage. The untrimmed mean of the data is included for comparison purposes only; it was not used for the analysis. The untrimmed mean exhibits substantially more high-frequency noise due to the effect of relative movement of the tracking region on border temperature pixels. As the majority of pixels in this area were part of the coolest 50% of values on any given frame, the use of the trimmed mean eliminates most of the tracking noise. As not all the border pixels are in the coolest 50%, the use of a low-pass filter on the trimmed mean removes remaining tracking noise due to higher temperature pixels near the border of the tracking region, as well as noise attributable to the thermal camera itself.

**Figure 3:**
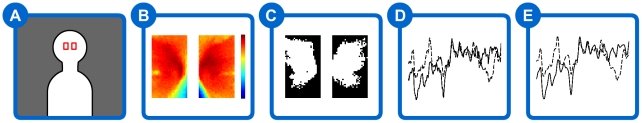
Mean temperature time series extraction procedure. **A:** The left and right periorbital regions are tracked from an initial reference frame to the end of the trial. **B:** The result of the tracking process is a pair of thermal videos, each showing one of the two periorbital regions (shown here with false colouring). **C:** For each frame of each periorbital's video, the temperature pixels are sorted from coolest to warmest and the warmest 50% of the pixels are identified. Note that the majority of the pixels on the border of the tracking region are not in the warmest 50%. **D:** For each frame, the mean of the 50% warmest pixels is taken for each periorbital region to generate a mean temperature time series for each periorbital region. **E:** The mean temperature time series is low-pass filtered to remove residual noise due to minor tracking fluctuations and thermal camera noise.

To further reduce high-frequency noise due to movement of the tracking region, as well as from the thermal camera itself, the mean temperature time series data were low-pass filtered using a second-order Butterworth filter with a cut-off frequency of 2 Hz. See Figure 2 for an illustration of the effects of these pre-processing techniques on typical data. The overall data extraction method is presented graphically in Figure 3.

### Data Analysis

Intervals of the mean temperature time series for each region were extracted pre- and post-stimulus presentation for each trial. The pre- and post-stimulus intervals were each two seconds in duration and were measured relative to the presentation of the auditory stimulus. A two second window length is justified as a previous investigation into the effect of the startle response on facial skin temperature indicated that a response occurs within 300 milliseconds [19], [20].

The pre- and post-stimulus intervals were compared using the following techniques:

1. Summary statistics

The range, mean, variance, skewness, and kurtosis of the mean temperature data from the pre- and post-stimulus intervals were compared using a three-way ANOVA with repeated measures for each participant. Grouping variables were pre- or post-stimulus, left or right periorbital, and participant. Although there is no physiological rationale for expecting a difference between the mean temperature time series of the left and right periorbital regions, this factor was included for completeness. Participant was treated as a random effect in the model (since all participants were assumed to represent the population from which they were drawn). Both the pre- and post-stimulus and left or right periorbital factors were treated as fixed effects.

2. Entropy

To test for differences in the information content of the mean temperature time series between the pre- and post-stimulus windows, the Shannon entropy [26] was computed. For each set of sample data, a histogram with 1000 equally spaced bins between the minimum and maximum sample values was generated. Each bin's count was normalized by the number of data points in the sample to produce an estimate of the probability mass function 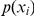 of that sample (where 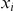 denotes the data point on which bin 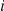 is centred). The entropy of the sample was then calculated by evaluating 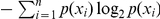, where 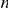 is the number of bins. Entropy was compared between the pre- and post-stimulus windows using the same method employed for the summary statistics of the mean temperature data. The inclusion of entropy in the analysis was motivated by a desire to examine the inherent disorder of the mean temperature time series data before and after the presentation of the stimulus.

3. Normality

The normality of the mean temperature time series within the pre- and post-stimulus windows for all trials was examined using the chi-squared goodness-of-fit test.

4. Stationarity (Entire Duration of Stimulus and Non-stimulus Trials)

The mean temperature time series was assessed for stationarity across the entire duration of each trial using the runs test [27] at the 5% significance level. This test was conducted to examine if the presence of an auditory startle stimulus affects the stationarity of stimulus trials as a whole compared to trials in which a stimulus was not presented.

#### Trials with a Visible Occurrence of the Formal Startle Response

To account for the possibility that, despite the temporal separation of sessions, participants may have habituated to the stimulus within or even possibly across sessions, the data were reanalyzed for the subset of stimulus trials in which the formal startle response was present (as verified by the procedure below). This prevents stimulus trials in which the participant was not startled or only mildly startled from masking the potentially more significant effect of trials in which a high magnitude, formal startle response did occur.

To identify stimulus trials in which a formal startle response visibly occurred, the thermal video was examined for the presence of sudden movements in a 3-second window after stimulus presentation. This is a conservative method of identifying formal startle responses, as some individuals can be startled in the formal sense but not exhibit involuntary movement.

## Results

Of the 1100 trials (50% with stimulus) collected across 11 participants, a total of 31 were unusable (16 stimulus and 15 non-stimulus). The unusable trials typically contained excessive participant movement that prevented the periorbital regions from being tracked. Of the 534 usable stimulus trials, 94 were deemed to contain a visible formal start response by the method introduced above.

### Summary statistics and entropy

Group means of the summary statistics and entropy measure for all stimulus trials are given in Table 1, organized according to the levels of the fixed effect ANOVA factors. There were no significant differences (p≥0.25) between the pre- and post-stimulus intervals or between the two regions for any of the statistical or entropy measures. Collectively, these results suggest that the stimulus had no effect on the statistical distribution of the mean temperature data of the periorbital regions.

**Table 1:** Group means of statistical and entropy measures for all stimulus trials (N = 534).

Measure	Pre- or Post-stimulus		Left or Right Periorbital	
	Pre	Post	Left	Right
Range (K)	0.050±0.023	0.051±0.022	0.051±0.023	0.051 ±0.023
Mean (K)	307.45±0.39	307.44±0.39	307.49±0.41	307.40±0.37
Variance (K^2^)	0.00030±0.00052	0.00030±0.00037	0.00030±0.00038	0.00030±0.00051
Skewness	−0.022±0.58	−0.0029±0.57	−0.0088±0.56	−0.016±0.59
Kurtosis	2.25±0.78	2.25±0.72	2.24±0.70	2.26±0.80
Entropy (bits)	4.91±0.053	4.92±0.048	4.92±0.051	4.92±0.051

### Normality

The vast majority of mean temperature data segments from both the left and right periorbital regions did not violate the null hypothesis of normality at a 5% significance level for either the pre- or post-stimulus interval (data from ≥98.5 of trials in all conditions were normally distributed).

### Stationarity

All trials in the four categories of left stimulus, left non-stimulus, right stimulus, and right non-stimulus were found to be non-stationary via the runs test. This corroborates previous work that characterized the skin temperature of the periorbital regions (among others on the face) as being largely non-stationary in nature [14].

### Trials with a Visible Occurrence of the Formal Startle Response Only

Group means of the summary statistics and entropy measure for the subset of stimulus trials with a formal startle response are shown in Table 2, organized according to the levels of the fixed effect ANOVA factors. The results for this subset of trials are similar to those for the full set of stimulus trials with the exception of a significant difference between the pre- and post-stimulus ranges and a significant difference between the means of the left and right periorbitals. Note that stationarity and normality were not retested for this subset of stimulus trials given that data from all trials were previously found to be non-stationary and the majority (≥98.5%) were normally distributed.

**Table 2:** Group means of statistical and entropy measures for the subset of stimulus trials containing a formal startle response (N = 94).

Measure	Pre- or Post-stimulus		Left or Right Periorbital	
	Pre	Post	Left	Right
Range (K)	0.055±0.034*	0.062±0.032^*^	0.059±0.032	0.059±0.035
Mean (K)	307.54±0.37	307.53±0.37	307.57±0.37^*^	307.50±0.36^*^
Variance (K^2^)	0.00040±0.0011	0.00048±0.00068	0.00044±0.00069	0.00044±0.0011
Skewness	−0.030±0.61	0.041±0.58	0.018±0.57	−0.0068±0.62
Kurtosis	2.27±0.81	2.27±0.78	2.21±0.70	2.33±0.87
Entropy (bits)	4.92±0.051	4.92±0.047	4.92±0.050	4.92±0.049

Statistically significant (p<0.05) differences between a pair of group means are denoted by a^*^ on both members of the pair.

## Discussion

### Periorbital Temperature Stability

**Figure 4:**
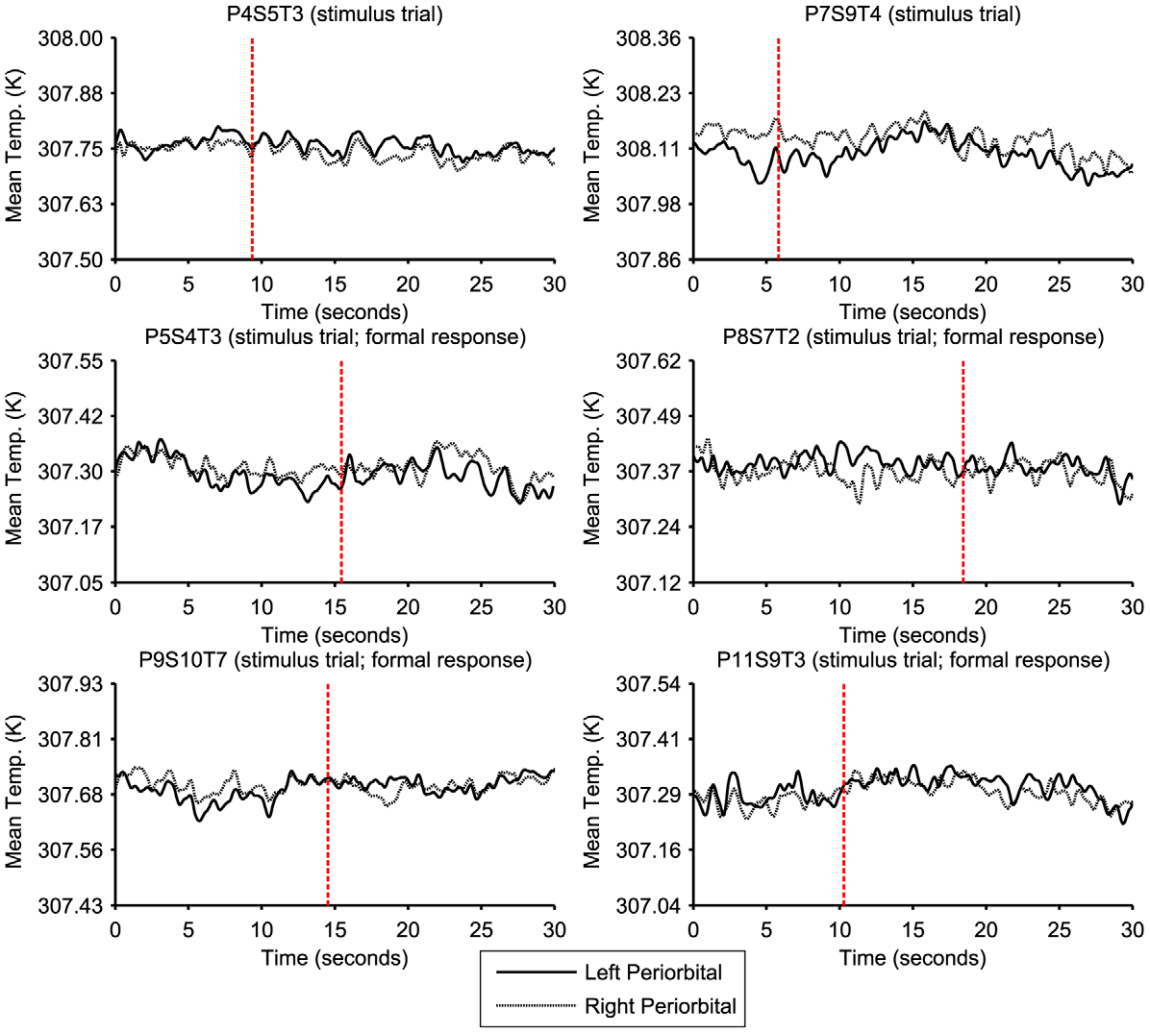
Plots of typical data taken from a selection of the stimulus trials. All vertical axes have been set to cover a range of 0.5 Kelvin. Note that the two plots in the first row are from stimulus trials in which a clear occurrence of the formal startle response was not present while the remainder are from those in which a formal response was identified. The vertical line denotes the time at which the unexpected auditory stimulus was presented.

Statistical analysis of the mean temperature data indicates that there is no significant change in the temperature of the inner canthi of the periorbital regions following the presentation of an unexpected auditory stimulus. The absence of a significant effect also held for only those trials in which a formal startle response was visibly evident. The quantitative results were supported by visual examination of the mean temperature time series data. Note that in the representative plots shown in Figure 4 the data remain within a relatively narrow range throughout the trials and appear unaffected by the stimulus.

Interestingly, these results contradict those reported by Pavlidis et al., who found that a warming of the periorbital regions occurred within 300 milliseconds of the presentation of a 60 dB stimulus [19]. This discrepancy could be explained by a number of differences between Pavlidis et al.'s work and the current study. In [19], the thermal equipment recorded non-radiometric color-mapped pixels to video (values between 81°F and 95°F were mapped to values between 0 and 255) and thus actual temperature values were not available. Furthermore, unlike the continuous temperature time series available in the present study, Pavlidis et al. presented data from only one point in time before and one point after stimulus presentation and it was not indicated when exactly the data points were taken relative to the time of stimulus presentation. It is also unclear whether the intensity values were taken from a single pixel within a given region or if they were the result of a spatial average over all the pixels in a region as in the current study. The statistical significance of the reported temperature differences was not assessed. It also appears from images included in [19] and Figure 2 of [20] that participants were not directly facing the thermal imaging device during the experiment and that the orientation of participants' faces relative to the thermal camera may have changed between the two points at which the pre- and post-stimulus pixel values were taken. Thermal imaging is known to be sensitive to viewing angle and specular reflections from ambient thermal radiation. In particular, viewing a surface at an oblique viewing angle will increase the proportion of incoming thermal radiation attributable to environmental sources and reduce the effective emissivity of the surface being viewed [28]. The apparent use of an oblique viewing angle in [19] (with a possible change in the angle between pre- and post-stimulus conditions due to participant movement, as shown in Figure 2 of [20]) could have had a non-negligible effect on the apparent temperature of the regions examined. It should also be noted that a 60 dB stimulus would be perceived to be approximately 16 times quieter than the stimulus used in the present study and there is no indication in [19] as to whether or not participants were startled in the formal sense. The above methodological differences likely account for the discrepancy between our results and those reported previously in the literature [19], [20].

### Statistical Characteristics

The mean temperature time series for both periorbital regions was non-stationary across the entire trial duration for non-stimulus trials. This result is consistent with previous research that has shown that mean facial skin temperature in the periorbital, nasal, and forehead regions is non-stationary [14]. The normality of the distribution of temperature values for the left and right periorbitals both pre- and post-stimulus windows indicates that they can likely be parameterized by their first two moments (mean and variance) alone.

For the set of all stimulus trials, summary statistics of the mean temperature data were not significantly different between the pre- and post-stimulus windows. When considering only those stimulus trials in which a formal startle response occurred, the range in the pre-stimulus window was significantly higher than in the post-stimulus interval. However, the actual difference between the means of the range pre- and post-stimulus was on the order of a hundreth of a Kelvin. A difference of this magnitude being marked as significant in this context is almost certainly attributable to chance, an interpretation strengthened by the fact that the standard deviation of the mean of the range is almost three times the magnitude of this difference (see the pre- and post-stimulus group means for range in Table 2). Even if such a change were real and consistent, it would be clinically negligible in the context of a thermal access system; changes in temperature accompanying variations in mental state would be on the order of at least a tenth of a Kelvin.

Although not relevant to the focus of this study, it should be noted that the statistically significant difference in the mean for the left and right periorbitals for the subset of trials containing a visible startle response is attributable to the fact that this subset happened to contain more trials in which the right periorbital was cooler than the left periorbital (i.e., the difference is attributable to chance). Such an occurrence is unsurprising given that the temperature distribution of the experimental environment could not, realistically, have been completely uniform. This factor, in combination with a certain degree of physiological asymmetry, likely accounts for the disparity between the mean temperatures of the regions of interest across the subset of trials with a visible startle. It should also be noted that this disparity is not of a sufficient magnitude to be clinically significant in the context of thermal access.

For both the set of all stimulus trials and the set including only those in which a formal startle response was present, there was no significant difference between the pre- and post-stimulus entropy for either periorbital region, indicating that the information content inherent in the mean temperature time series was unaffected by the presentation of the stimulus regardless of the magnitude of the associated response.

### Physiological Interpretation

It is rather surprising that no change in the mean temperature was associated with the presentation of a loud, unexpected auditory stimulus, particularly for trials in which it was clear that the formal startle response did occur. The underlying physiology of the startle response is intrinsically linked to action of the sympathetic nervous system and adrenal medulla in the context of the so-called “fight-or-flight” reaction, which is manifested in response to environmental stressors. As part of this response, sympathetic nerve fibres trigger an increase in the rate and strength of cardiac contractions and localized elevations in blood pressure, heightening blood flow to the musculoskeletal system and other essential tissues. The release of epinephrine by the adrenal medulla triggers, among other responses, a flattening of the lenses of the eyes (optimizing far vision) and dilation of the pupils [29].

Action of the sympathetic nervous system is known to influence perfusion of the face, particularly in the context of stressors such as embarrassment or discomfort [10] and the eye itself is surrounded by extensive vasculature. Of particular relevance to this study are the angular artery and the angular vein, both of which run underneath the inner canthus of the periorbital region of their associated eye [30]. The angular artery supplies the orbicularis oculi muscle, the activity of which is frequently measured for the purposes of detecting occurrences of the formal startle response [23].

These physiological factors point strongly towards increased perfusion in the vasculature surrounding the eye as a component of the startle response. However, given the results described herein, if an increase in perfusion is associated with the startle response, it does not create a significant change in temperature. Although unexpected, this is quite plausible for lower magnitude (i.e., non-formal) startle responses. For formal startles (in this study, those that resulted in a generalized and obvious musculoskeletal response in the upper body), it is possible that perfusion is increased but blood flow is still sufficiently localized such that no significant temperature change occurs on the surface of the inner canthus of the periorbital region. Given that core body temperature is generally around four Kelvin higher than surface temperature [31], it is logical to expect that the increased cardiac output associated with activity of the sympathetic nervous system would result in a temperature increase. However, as the majority of blood flow during a fight-or-flight reaction is directed to the musculoskeletal system [29], it is possible that any increased perfusion of the ophthalmic complex would draw on blood relatively close to the ocular vasculature and thus already at a similar temperature.

### Tracking

The tracking method used in this study (the cross-search algorithm with MSE as the matching criterion), although effective in this particular context, would not be a practical choice for real-world applications. In this experiment, participants were explicitly instructed to remain relatively immobile and the only substantial movement that occurred in usable trials was the generalized muscular response associated with the formal startle response. This type of movement did not involve substantial rotation of the head or changes in position that might have occluded one or both periorbital regions and thus did not impair tracking performance. In fact, any block-matching algorithm would likely have been sufficient for this application; the cross-search algorithm was chosen for its speed and computational simplicity [24].

In a clinical application where the user may retain some movement, either voluntary or involuntary, a more robust tracking procedure (such as the one detailed in [21]) might be necessary. This need was highlighted by trials in the present study that were unusable because the tracking algorithm failed when the participant moved his or her head such that one or both of the periorbital regions left the camera's field of view or became occluded. This suggests that, in an access context, multiple thermal imaging devices positioned at different angles may be required to ensure that the user's face remains in view.

However, for a thermographic access system aimed at individuals with total LIS, the difficulties associated with reliable tracking would likely not exist. As these individuals have no capacity for voluntary movement, a thermal access system for this client population might operate effectively without the use of a tracking algorithm at all; simply indicating the location of the regions of interest on the user's face could be sufficient.

### Implications for Access Technology Design

Overall, this study indicates that a loud, unexpected auditory stimulus does not result in a significant change in the mean temperature of the inner canthi of the periorbital regions, even when the stimulus triggers a formal startle response in the individual perceiving it. Although the startle response can be triggered by non-aural stimuli, the response itself is not known to be dependent on the type of stimulus and, as such, the results of this study should extend to all occurrences of the startle response, not simply those triggered by auditory stimuli. As absolute changes in temperature have been identified as a promising basis for an access system based on thermography [11], this is a favourable result. These findings suggest that the occurrence of false positive activations due to startling events in the environment is not a concern in the development of a thermography-based access technology.

The apparent insensitivity of thermography of the periorbital regions to startle responses is an important advantage over other technologies, such as EEG and NIRS [12], [32], currently being investigated as access systems for individuals with total LIS. While these technologies can provide a more direct indication of mental state, the present findings combined with the previously established link between facial skin temperature and emotional state suggest that thermography has potential to improve caregiver interpretation of individuals with total LIS. Additional research into the effects of deliberate modulation of one's emotional state on thermal data will be required prior to clinical introduction.
